# Toxin–Antitoxin Systems in *Bacillus subtilis*

**DOI:** 10.3390/toxins11050262

**Published:** 2019-05-09

**Authors:** Sabine Brantl, Peter Müller

**Affiliations:** Friedrich-Schiller-Universität Jena, Matthias-Schleiden-Institut, AG Bakteriengenetik, Philosophenweg 12, D-07743 Jena, Germany; p.mueller@uni-jena.de

**Keywords:** toxin–antitoxin system, antisense RNA, small regulatory RNA (sRNA)/target RNA interaction, toxic peptide, RNA degradation, prophage

## Abstract

Toxin–antitoxin (TA) systems were originally discovered as plasmid maintenance systems in a multitude of free-living bacteria, but were afterwards found to also be widespread in bacterial chromosomes. TA loci comprise two genes, one coding for a stable toxin whose overexpression kills the cell or causes growth stasis, and the other coding for an unstable antitoxin that counteracts toxin action. Of the currently known six types of TA systems, in *Bacillus subtilis*, so far only type I and type II TA systems were found, all encoded on the chromosome. Here, we review our present knowledge of these systems, the mechanisms of antitoxin and toxin action, and the regulation of their expression, and we discuss their evolution and possible physiological role.

## 1. Introduction

Toxin–antitoxin (TA) systems are genetic modules that are widespread in bacterial genomes and plasmids. They consist of a gene pair encoding a stable toxin that impedes cell growth by interfering with fundamental processes, and an unstable antitoxin that counteracts the toxin activity. Currently, six types of TA systems are known. Whereas type I and III TA systems employ RNA antitoxins, the other TA systems use proteinaceous antitoxins. In type I TA systems, antitoxins interact with the corresponding toxin messenger RNAs (mRNAs) [1]. In type III systems, the RNA antitoxin binds the toxin protein directly. By contrast, type II antitoxins inhibit their toxins via a protein/protein interaction. Antitoxins in type IV TA systems interfere with binding of the toxin proteins to their cellular targets [2]. In type V systems, the antitoxin is an RNase which specifically cleaves the toxin mRNA, thus preventing toxin expression [3]. In the recently discovered type VI systems, the toxin binds directly to the β sliding clamp DnaN to block replication elongation, whereas the antitoxin promotes degradation by the ClpXP protease [4]. The toxin targets are diverse and comprise DNA replication, stability of mRNA or DNA, protein synthesis, cell-wall biosynthesis, membrane integrity, and ATP synthesis. 

Although TA systems are ubiquitous in bacterial genomes and their molecular activities were investigated for many years, the biological function of the majority of them is still elusive. To date, the following major biological functions were demonstrated: Post-segregational killing (PSK), abortive infection, persister formation/antibiotic tolerance, and stress response (reviewed in Reference [5]). 

In *Bacillus subtilis*, both type I and type II TA systems were discovered and characterized. In this review, we provide an overview of the known *B. subtilis* TA systems with special focus on their molecular mechanisms, the control of toxin and antitoxin expression, the modes of toxin action, and their biological role.

## 2. Type I Toxin–Antitoxin Systems

### 2.1. Overview of *B. subtilis* Type I TA Systems

In 2005, the first type I TA system was discovered in *B. subtilis* and designated *txpA*/RatA [6]. Subsequent transcriptomics [7] and bioinformatics studies [8] revealed 14 potential type I TA systems [9], five of which are located on prophage regions. Four of them—*txpA*/RatA [6,10], *bsrG*/SR4 [11], *bsrE*/SR5 [12,13] and *yonT-yoyJ*/SR6 [14]—were investigated in more detail (Table 1) and are reviewed here. The other nine potential systems, the putative toxins of which were grouped into the YhzE family [9], are yet to be confirmed as TA systems.

### 2.2. Mechanisms of Antitoxin Action

**Figure 1:**
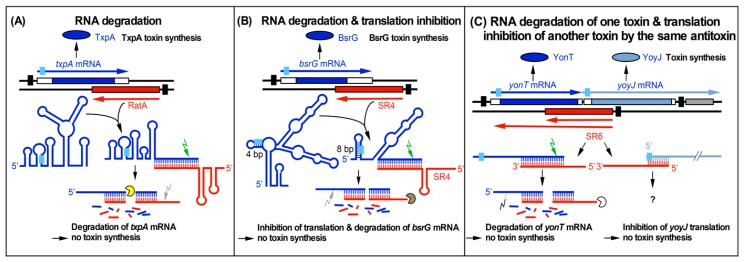
Three currently known mechanisms of action used by *Bacillus subtilis* type I antitoxins. Black bars denote promoters. Toxins are drawn in blue or blue-gray and antitoxins in red. The toxin open reading frames (ORFs) are represented by blue and blue-gray bars. Light-blue boxes denote ribosome binding sites (RBS). Arrows symbolize endoribonucleases (RNase III, green; RNase Y, gray; white, unknown RNase) and circular segments symbolize 3′–5′ exoribonucleases (PNPase, yellow; RNase R, brown; unknown RNase, white). (**A**) Promotion of RNA degradation. The antitoxin RatA and its *txpA* toxin messenger RNA (mRNA) base-pair at their 3′ ends. (**B**) RNA degradation and translation inhibition. The antitoxin SR4 and the corresponding *bsrG* toxin mRNA interact at their 3′ ends. SR4 binding to *bsrG* mRNA induces a conformational alteration that extends the region sequestering the Shine Dalgarno (SD) sequence from 4 bp to 8 bp which inhibits *bsrG* translation. Additionally, the SR4/*bsrG* mRNA interaction facilitates toxin mRNA decay by an initial Rnase III cleavage followed by subsequent RNase R and RNase Y degradation. (**C**) One antitoxin inhibits two toxins via different mechanisms. Antitoxin SR6 and *yonT* toxin mRNA base-pair at their 3′ ends, which promotes *yonT* mRNA decay via an initial RNase III cleavage that is followed by degradation by so far unidentified RNases. Furthermore, SR6 interacts with *yoyJ* toxin mRNA by base-pairing at the 5′ ends, which does not promote *yoyJ* mRNA degradation, but prevents *yoyJ* overexpression, most likely via translational inhibition. (**A**,**B**) are based on Reference [18].

Two principal mechanisms of action of type I antitoxins are known: inhibition of toxin translation or promotion of degradation of the toxin mRNA. *B. subtilis* SR4 is the first bifunctional antitoxin; it both impedes translation of *bsrG* mRNA and facilitates its degradation [15] (Figure 1). 

In cases of *Escherichia coli* type I TA systems, where the complementarity between antitoxin and toxin mRNA resides in their 5′ regions, the antitoxin either binds at a region overlapping the toxin Shine Dalgarno (SD) sequence to inhibit toxin translation directly (e.g., *E. coli* SymR), or it prevents translation of a leader peptide translationally coupled to the toxin (e.g., *hok*/Sok or *ldrD*/RdlD [16]). Alternatively, the antitoxin binds to a ribosome standby-site to block toxin translation (e.g., *E. coli tisB*/IstR1 [17]).

By contrast, in the *B. subtilis* type I TA systems studied so far (*txpA*/RatA [6], *bsrG*/SR4 [11], *bsrE*/SR5 [12], *bsrH*/as-bsrH [10], and *yonT*/SR6 [14]), the RNA antitoxins bind at the 3′ end of the toxin mRNAs and support their degradation. This was demonstrated in four instances by the longer half-lives of the toxin mRNAs in the absence of their corresponding antitoxins [10,11,12,14]. In these cases, RNA antitoxins and their toxin mRNAs share a rather long complementary stretch of 100 to 140 bp. For *bsrG*/SR4, it was found that SR4 not only promotes degradation of *bsrG* mRNA, but induces a conformational change around the *bsrG* RBS that further impairs ribosome binding, thus additionally impeding toxin translation [15]. Neither upon RatA binding to *txpA* mRNA [10] nor antitoxin SR5 binding to *bsrE* mRNA [13] was such a conformational change observed, suggesting that RatA and SR5 only cause toxin degradation.

For the *yonT*/SR6 TA system, no secondary structures were probed to investigate this issue. Interestingly, SR6 controls a second toxin gene, *yoyJ*, using its 5′ region to base-pair to the *yoyJ* ribosome binding site (RBS) [14]. Whereas SR6 promotes *yonT* mRNA degradation, it neither affects the amount nor half-life of *yoyJ* mRNA [14]. Therefore, it seems to inhibit *yoyJ* translation. 

### 2.3. Binding Pathway of RNA Antitoxin and Toxin mRNA

**Figure 2:**
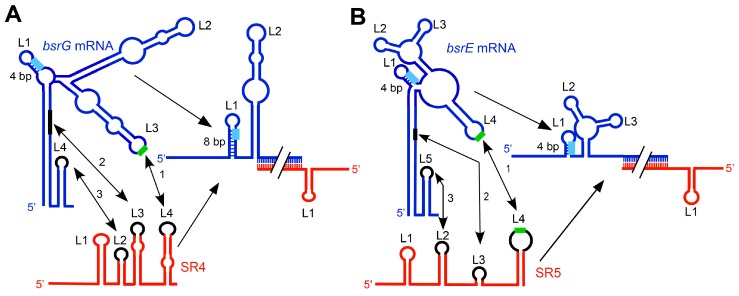
Comparison of the SR4/*bsrG* RNA (**A**) and SR5/*bsrE* RNA (**B**) interaction pathways. Blue, toxin mRNAs; red, RNA antitoxins. U-turn motifs are indicated by green and SD sequences by light-blue boxes. The interaction chronology is designated by 1 to 3; L, loop. (**A**) The initial contact between SR4 and *bsrG* RNA takes place between L4 of SR4 and L3 of *bsrG* RNA (1). It is followed by helix progression to an interaction between SR4 loop L3 and the 3′ part of helix P1 of *bsrG* RNA (2), and finally reaches L2 of SR4 that binds terminator loop L4 of *bsrG* RNA (3). The latter interaction is not essential. (**B**) The binding pathway of SR5 and *bsrE* RNA comprises three similar subsequent interactions. The schematic secondary structures are based on the experimentally probed structures [13,15]. (**A**) is based on Reference [18].

Binding pathways were studied in detail for many *cis*-encoded sense/antisense systems, and binding kinetics of sense and antisense RNAs were measured (reviewed in Reference [19]). On average, *k*_app_ values (apparent pairing rate constants) are in the range of 1 × 10^5^ to 1 × 10^6^ M^−1^·s^−1^. High *k*_app_ values are indicative for efficient pairing between two complementary RNAs. For *bsrG*/SR4, a *k*_app_ value of 6.5 × 10^5^ M^−1^·s^−1^ was determined [15] and, for *bsrE*/SR5, a *k*_app_ value of 4 × 10^6^ M^−1^·s^−1^ [13]. For *txpA*/RatA and for *yonT*/SR6, *k*_app_ measurements are yet to be performed. To narrow down the minimal regions required for efficient complex formation with their toxin mRNAs, truncated SR4 or SR5 species were employed in interaction studies with their toxin mRNAs [13,15]. Both wild-type RNA antitoxins are composed of four stem-loops (SLs) (see Figure 2). An SR4 species composed of only SL 4 (terminator SL) and the 3′ arm of SL 3 was still perfectly able to bind its target [15]. By contrast, both SL 2 and SL 4 of SR5 were found to be essential for formation of a stable duplex with *bsrE* RNA, since replacement of either of them or of both loops 2 and 4 decreased binding 6–7-fold [13]. 

Initial contacts between a base-pairing small regulatory RNA (sRNA) and a target mRNA can either take place between two complementary loops (e.g., *fst*/RNAII [20]) or between a loop and a single-stranded region (e.g., *hok*/Sok [21]). In both pathways, the interaction always yields a complete duplex that is cleaved by the double-strand specific RNase III. 

In the case of *bsrG*/SR4, binding starts between the so-called recognition loops, the terminator loop L4 of SR4 and L3 of *bsrG* RNA, followed by base-pairing between L3 of SR4 and a stretch within the main helix of *bsrG* RNA. Eventually, SR4 L2 and the *bsrG* L3 interact, although this interaction is not required for inhibition [15] (Figure 2A). A similar binding pathway including three consecutive interactions was elucidated for *bsrE/*SR5 ([13] Figure 2B). However, three differences to *bsrG*/SR4 can be pointed out. Firstly, whereas, in *bsrG*/SR4, an antitoxin comprising only SL 4 and the 3′ half of SL 3 is sufficient for inhibition, two loops of SR5 are necessary for successful interaction with *bsrE* RNA. Secondly, two U-turn motifs (see below) are involved in the first loop–loop contact, whereas only one U-turn motif participates in the *bsrG* RNA/SR4 interaction. Thirdly, in contrast to SR4, SR5 does not induce structural changes around the *bsrE* SD, i.e., the stem topped by L1 is not extended.

In the case of *txpA*/RatA, interaction most probably also initiates via a loop/loop contact, but the binding pathway is yet to be investigated in detail [10]. By contrast, a concurrent interaction of two loop pairs was observed for the TA system *sib*/Ibs of *E. coli* [22], as well as earlier for RNAII/III, the replication control system of the streptococcal plasmid pIP501 [23]. 

In sense/antisense RNA systems, a rapid interaction between both RNAs is crucial for efficient regulation. A 5′ YUNR motif (Y, pyrimidine; N, any base; R, purine) can potentially form a U-turn with a sharp bend in the RNA backbone that presents the next three or four bases in a stacked, solvent-exposed configuration. This provides a scaffold for rapid RNA/RNA interactions. Via one or two contacts across the bend, the backbone change is stabilized. U-turn motifs were first discovered in yeast transfer RNA (tRNA) and the hammerhead ribozyme—and confirmed by crystal structure analysis—and later in 45 sense or antisense RNAs (reviewed in Reference [24]). *E. coli* type I TA system *hok*/Sok was the first sense/antisense RNA system in which a 5′ YUNR motif present in the sense (*hok*) mRNA was demonstrated to be decisive for a rapid RNA interaction with the Sok antisense RNA [25]. Later, this was also shown for RNAII/RNAIII of pIP501 [26]. Only in these two systems was U-turn formation experimentally confirmed by *N*-ethyl, *N*-nitrosourea (ENU) footprinting. Surprisingly, in the case of *bsrG*/SR4, two potentially important 5′ YUNR motifs were detected. However, whereas the motif in L3 of *bsrG* RNA is implicated in the initial contact with L4 of SR4, the 5′ YUNR motif in L2 of SR4 that interacts with *bsrG* L4 was not required, as the entire SL2 was not essential for the inhibitory function of SR4 [15]. In *bsrE*/SR5, two loops (L4 of *bsrE* RNA and L4 of SR5) each containing a U-turn motif are involved in the initial rapid interaction ([13] Figure 2B). By contrast, in *txpA*/RatA, neither of the interacting loops contains a 5′ YUNR motif [10]. So far, secondary structures are yet to be determined experimentally for *yonT-yoyJ*/SR6 and *bsrH*/as-bsrH. Therefore, it is unknown if 5′ YUNR motifs are present in loop regions of either toxin mRNA or RNA antitoxin.

### 2.4. Additional Modes to Preclude Premature Toxin Expression

Additional strategies that utilize the high capacity of RNA to fold and refold are employed by the different TA systems to ensure tight regulation of toxin expression. Whereas in Gram-negative bacteria, processing events can convert long, inactive toxin mRNAs into short, translationally competent ones (reviewed in Reference [18]), alternative approaches were found in *B. subtilis*. The toxin genes *txpA, bsrE*, and *bsrG* sequester their ribosome binding sites (RBS) in four or five GC base-pairs [9,13,15], which suffice to prevent efficient binding of the 30S ribosomal subunit. For *bsrG*, in vitro translation products could be only obtained after mutations were introduced to open the double-stranded region in the mRNA [15]. An alternative strategy was found for *txpA* and *yonT*, whose RBSs display more than 11 bp complementarity to the anti-SD in 16S rRNA. Such perfect RBSs are predicted to allow efficient recruitment, but only slow release of ribosomes (reviewed in Reference [9]). *B. subtilis yonT*—as *Enterococcus faecalis fst*—contains the rare start codon GUG that is found instead of AUG in only 14% of all genes in *B. subtilis*. This is expected to reduce translation efficiency. In other type I TA systems from Gram-positive bacteria, compact secondary structures comprising RNA pseudoknots were proposed to impede internal ribosome binding (reviewed in Reference [18]).

### 2.5. Role of Hfq

Hfq is an abundant RNA chaperone that was found—among other things—to either stabilize *trans*-encoded base-pairing small regulatory RNAs (sRNAs) or to promote the interaction with their target mRNAs [27,28]. The latter function is particularly important when both interacting RNAs share only a few complementary base-pairs. Among the currently known type I TA systems, this is only the case for the *E. coli* TA system *ralR*/RalA with a short (16 bp) stretch of complementarity between *ralR* mRNA and its antitoxin RalA. Here, indeed, a requirement of Hfq was discovered [29]. Neither the other *E. coli* antitoxins nor the *B. subtilis* antitoxins like SR4, SR5, or SR6 [11,12,14], which display ~120-nt complementarity to their toxin mRNAs, need Hfq. Although Hfq stabilizes SR5 about seven-fold, it was not required for the inhibitory function of this antitoxin [12]. Interestingly, it was recently shown in *B. subtilis* that the widespread RNA chaperone CsrA whose recognition sequence comprises a 5′GGA motif in a single-stranded or loop region can also restructure an mRNA to facilitate the interaction with its regulatory sRNA [30]. Therefore, it is not excluded that CsrA or other, still unidentified RNA binding proteins might accomplish the function of Hfq for toxin mRNA/antitoxin pairs with very short base-pairing regions.

### 2.6. RNases Involved in Antitoxin/Toxin mRNA Degradation

In the majority of *cis*-encoded sRNAs, RNase III, the double-strand specific RNase present in all bacteria, cleaves the antisense/sense RNA duplex to initiate target RNA degradation resulting in inhibition of gene expression. This was discovered 30 years ago for antisense RNAs encoded on plasmids, transposons, and bacteriophages (reviewed in Reference [18]). In type I TA systems, RNase III was found to cleave the *hok* toxin mRNA/Sok antitoxin duplex from *E. coli* plasmid R1 [31], and >20 years later the *txpA*/RatA [10], *bsrG*/SR4 [11], and *bsrE/*SR5 [12] duplexes (Table 1). Therefore, in some cases, RNase III is essential to inhibit the toxin mRNA, e.g., for RatA, SR6 (formerly as-yonT [10]), and *E. coli* OrzO [32]. By contrast, it is not required by *B. subtilis* SR4 [11], as well as the *E. coli* antitoxins Sok (reviewed in Reference [18]), SymR (reviewed in Reference [16]), IstR1 (reviewed in Reference [33]), and Sib (reviewed in Reference [34]). Apparently, RNase III is only necessary in cases where target control is accomplished exclusively via mRNA degradation. However, in cases where RNA antitoxins regulate steps preceding degradation like translation (SymR, Sok, IstR1), RNase III is dispensable and only eventually cleaves the antitoxin/toxin mRNA duplex. 

A comparison between *txpA*/RatA and *bsrG*/SR4 from *B. subtilis* corroborates this hypothesis. SR4 is a bifunctional antitoxin that not only facilitates *bsrG* RNA degradation, but also impedes *bsrG* translation [15]. Neither cell lysis nor mutations in the *bsrG* ORF were observed in an *rnc* knockout strain [11] demonstrating that RNase III is not essential for toxin inhibition. RNase III cleaves *bsrG* mRNA 14 nt downstream of the 3′ end of complementarity with SR4 at position 185. By contrast, RNase III cleavage of *txpA* RNA in its duplex with RatA is essential to prevent toxin expression [10]. RatA only decreases target mRNA stability by recruiting RNase III. However, as was previously shown by in vitro secondary structure probing [10], it does not induce a *txpA* mRNA conformation that further inhibits translation. In the absence of RNase III, *txpA* RNA levels were six-fold enhanced, whereas those of RatA were not affected. RNase III is also essential for *yonT* RNA degradation, which shows a seven-fold higher stability in the absence of RNase III [10]. For *bsrE*/SR5, two- to three-fold higher amounts of *bsrE* RNA were found in the absence of RNase III in the logarithmic and stationary phase, respectively [12]. Three distinct cleavage sites of RNase III were mapped near the *bsrE* stop codon [12]. However, as BsrE is a much weaker toxin than BsrG, and only *bsrE* overexpression inhibits cell growth and causes cell lysis, RNase III was not found to be essential [12]. 

The role of RNase III for antitoxin stability differs between the various *B. subtilis* type I TA systems. SR5 [12] and SR6 [10] behave like RatA in that their half-lives are almost not affected by RNase III, whereas SR4 displays a 2.5-fold higher stability in an *rnc* mutant strain [11]. Apparently, the relative amount of antitoxin and toxin mRNA is important. Both RatA [10] and SR5 [12] are in excess over their target RNAs. SR6 is in more than 10-fold excess over *yonT* mRNA in complex medium and minimal medium without glucose in all growth phases. Only in minimal medium with glucose, from late log phase toward stationary growth phase, were 3:1 to 1:1 SR6/*yonT* RNA ratios determined [14]. By contrast, SR4 is in excess over *bsrG* RNA only in complex medium from the transition phase until late stationary phase [35]. 

Surprisingly, in the case of *B. subtilis bsrH*/as-bsrH, the levels of *bsrH* toxin mRNA are neither affected by RNase III nor by RNase Y, but by RNase J1, an endonuclease and 5′–3′ exoribonuclease. Degradation was proposed to be rather exoribonucleolytic [10]. RNase J1 also participates in the degradation of antitoxin SR5 and *bsrE* mRNA [12], whereas its effect on *bsrG* RNA and SR4 was only 1.5-fold [11,35].

An influence of the main endoribonuclease RNase Y on the degradation of both SR4 and *bsrG* RNA was found. Whereas *bsrG* RNA was two-fold less stable in the absence of RNase Y, SR4 showed a two-fold higher stability [35]. The effect on *bsrE* RNA was similar to that on *bsrG* RNA, while RNase Y did not affect SR5 [12]. The higher stability of both *bsrG* and *bsrE* RNA in the absence of RNase Y might be due to indirect effects. RNase Y also initiates degradation of RatA. Afterwards, the 5′ part of RatA alone is degraded by 3′–5′ exoribonuclease PnpA and the 3′ part by 5′–3′ exoribonuclase J1 ([10] Figure 1).

RNase R is the main 3′–5′ exoribonuclease involved in the degradation of both *bsrG* mRNA and SR4. In addition, the 3′–5′ exonuclease PnpA trims both SR4 and SR5 precursors from their 3′ ends to the mature antitoxin, but only marginally contributes to degradation of both RNA antitoxins [11,12]. PnpA increases the stability of *bsrE* RNA [12], as, in its absence, two- to 10-fold lower amounts of *bsrE* RNA were observed in the stationary and logarithmic growth phase, respectively. Further processing of the RNase III cleavage products of *txpA* RNA is also performed by PnpA [10].

In summary, all *B. subtilis* type I toxin mRNAs—with the exception of *bsrH* mRNA—are cleaved by RNase III when they are base-paired to their RNA antitoxins. RNase III cleavage is, however, only required for inhibition when the corresponding antitoxins exclusively promote RNA degradation. Various other endo- and exoribonucleases act in concert to regulate antitoxin and toxin mRNA stability. 

### 2.7. Mode of Action of Type I Toxins

**Figure 3:**
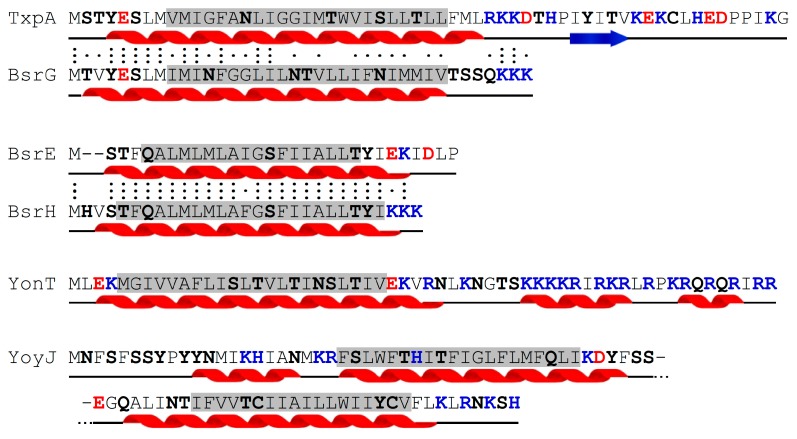
Overview of *B. subtilis* type I toxins. The amino acid (aa) sequences are shown with the predicted secondary structures (Jpred4) below. Red helices indicate α-helical and blue arrows β-sheet structures. A black line indicates unstructured regions. Transmembrane domains (predicted by TMHMM v. 2.0, DTU Health Tech, Lynby, Denmark) are highlighted in gray; polar aa are shown in bold, and charged aa are red (−) and blue (+). Conserved aa are indicated with three dots, and similar aa with one dot (.) between similar sequences. Based on Reference [9].

The vast majority of the currently known type I toxins are small hydrophobic peptides of less than 60 amino acids (aa) that have a similar predicted secondary structure containing an α-helical transmembrane domain (see Figure 3), which in some cases was experimentally confirmed. They are localized in the membrane/inner membrane and are suggested to create pore-like structures destroying the membrane potential and causing cell death by energy starvation (summarized in Reference [18]). Examples are the *E. coli* toxins Hok and TisB (reviewed in Reference [36]). Exceptions are the *E. coli* toxins SymE (113 aa), an RNase [37], and RalR, a DNase [29], which are both located in the cytoplasm. However, little is known about the mechanism of action and the targets of the membrane-bound hydrophobic type I toxins, predominantly due to the fact that phenotypical effects are often only detectable upon toxin overexpression. 

The *B. subtilis* type I toxins TxpA, BsrG, BsrE, and YonT reviewed here are smaller than 60 aa and contain transmembrane domains (Figure 3). Therefore, they are proposed to be inserted into the membrane. YoyJ is the only larger toxin (83 aa) and displays two transmembrane domains.

Whereas deletion of the antitoxins RatA or SR4 causes cell lysis by the cognate toxins TxpA or BsrG on agar plates after five days [6] or one day [11], respectively, this was not the case for deletion of antitoxin SR5 regulating *bsrE*. Only *bsrE* overexpression resulted in cell lysis and impaired growth [12]. In the case of *yonT-yoyJ*/SR6, it was not possible to delete the antitoxin gene or to overexpress the toxin gene, whether in *E. coli* or in *B. subtilis,* indicating a high level of toxicity. For YoyJ being a weak toxin, only indirect proof could be obtained. A *yoyJ* overexpression plasmid could only be established in *B. subtilis* in the presence of the *sr6* gene, but not in *B. subtilis* strain 1A100 that lacks the SPβ prophage region encoding *yonT-yoyJ/*SR6 [14]. 

BsrG is the only *B. subtilis* type I toxin that was studied in more detail [38]. In contrast to *E. coli* type I toxins Hok, TisB, and LdrD that kill their hosts within 30 min of induction [39,40,41], BsrG unfolds its toxic effect in growing cells via a gradual, relatively slow process. Although it is inserted into the membrane, it neither induces pores nor disrupts the membrane potential. By contrast, it causes membrane invaginations which lead to delocalization of the MreB cytoskeleton and the associated cell-wall synthesis machinery. This results in altered cell morphology, aberrant cell division sites, and finally, cell lysis. Furthermore, compaction of the nucleoid and inhibition of protein biosynthesis were observed [38]. In two independent transposon mutagenesis screens, it was impossible to identify a BsrG target (Jahn, unpublished observations), suggesting that it is an essential protein. 

Our data on the effects of BsrG question the simplistic assumption mentioned above that all membrane-targeting type I toxins act like phage holins and permeabilize the membrane. Interestingly, overproduction of the type I Fst toxin in *Enterococcus faecalis* yields misplaced septa, resulting in daughter cells containing little or no DNA, and the previously reported increase in membrane permeability was found to be a secondary effect, presumably caused by segregation defects [42]. This suggests that more type I toxins might act similarly to BsrG. 

### 2.8. Regulation of Toxin and Antitoxin Expression and Biological Role

For a tight control of toxin expression, the balance between the antitoxin and toxin mRNA levels is critical. High antitoxin/toxin mRNA ratios successfully prevent toxin expression and ensure cell survival. By contrast, under conditions where the toxin mRNA is in significant excess over the antitoxin, the toxin can escape repression and cause growth arrest or cell death.

Toxin expression can be regulated transcriptionally or post-transcriptionally, e.g., depending on stress conditions or the nutritional state of the cell [43]. An example for transcriptional control is SOS control found for three *E. coli* type I toxin genes, *symE*, *tisB*, and *dinQ*, which contain Lex boxes overlapping their promoters (reviewed in Reference [18]).

By contrast, temperature sensitivity described for the three *B. subtilis bsrG*, *bsrE*, and *yonT* RNAs [11,12,14] depends on RNA degradation, i.e., it is based on post-transcriptional control. The amount of *bsrG* and *bsrE* RNA decreases after heat shock at 48 °C or 55 °C, whereas the amount of *yonT* RNA increases. Both antitoxins SR4 and SR5 are not affected by heat shock, but the amount of SR6 decreases concomitantly with the increase of its toxin mRNA [14]. Refolding at 55 °C makes *bsrG* RNA more accessible to single-strand-specific RNases J1 and Y [35] resulting in a three- to four-fold shorter half-life at 48 °C or 55 °C compared to 37 °C [11,35]. As *bsrE* RNA has a similar secondary structure to *bsrG* RNA (compare References [13] and [15], Figure 2), it can most probably refold in a similar manner at high temperatures. The secondary structure of *yonT* mRNA is yet to be determined. Therefore, it is unclear whether a structural alteration or the lower abundance of SR6 under heat-shock conditions is responsible for the higher abundance of *yonT* mRNA.

Both *bsrE*/SR5 and *yonT*/SR6 are multistress-responsive TA systems [12,14]. The *bsrE* and the *yonT* toxin RNAs are extremely unstable upon ethanol stress, whereas their RNA antitoxins are much less affected. The instability of *bsrE* RNA is due to rapid degradation by RNase Y [12]. In addition, *bsrE* RNA is also influenced by alkaline stress, while the amount of its antitoxin SR5 is affected by various stresses, among them oxygen deficiency, pH stress, and iron limitation. Oxygen stress is the only condition under which SR5 is not in excess over *bsrE* RNA anymore to prevent toxin expression [12]. For *yonT* RNA and SR6, strong effects of vancomycin (cell-wall stress) and manganese stress were observed (four- to five-fold decreased amounts). Lower effects were found for *yonT* RNA and SR6 upon salt stress, iron limitation, and oxidative stress [14]. Only the iron limitation effects in both TA systems and the oxygen stress effect on SR6 are *sigB*-dependent, suggesting a transcriptional regulation. Most probably, *bsrH* mRNA is also multistress-responsive, as riboprobes used for the detection of *bsrE* mRNA also allowed *bsrH* mRNA detection in Northern blots (Müller, unpublished observation).

No difference between antitoxin expression in rich or minimal medium was observed for *B. subtilis* SR4 [11] and SR5 [12]. By contrast, SR6 is much higher expressed in complex medium compared to minimal CSE medium with or without glucose [14].

Whereas plasmid-encoded type I TA systems play a role in post-segregational killing, the biological role of chromosome-encoded type I TA systems, such as the homologs of *hok/*Sok in *E. coli* (e.g., Reference [44]) or *fst*/RNAII in *E. faecalis* [45], is largely unknown. 

Some chromosomally encoded type I TA systems are only induced under certain metabolic or environmental stress conditions. Therefore, they could play a role in metabolic or stress adaptation by, e.g., killing the cells when nutritional sources or oxygen are exhausted. For *bsrG*/SR4, a 6–8-fold excess of toxin mRNA over antitoxin in transition and early stationary growth phase in complex medium was calculated, suggesting a function in growth adaptation [35]. Furthermore, the *bsrG* toxin mRNA is temperature-sensitive and could play a role during temperature changes [11]. By contrast, BsrE might be important under anaerobic stress, as this is the only stress condition where *bsrE* toxin mRNA is in excess over its antitoxin SR5 [12], while, in unstressed cells and under heat shock, iron limitation and pH stress, SR5 is in >5-fold excess and can prevent toxin expression. For *yonT* RNA and SR6, strong effects of vancomycin (cell-wall stress) and manganese stress were observed. However, no relative ratios of SR6 and *yonT* RNA were calculated under stress conditions. In a transcriptomics study, the amounts of *txpA* and *bsrH* mRNA were found to increase after exhaustion of glucose [46], suggesting a role for TxpA and BsrH under nutritional stress.

### 2.9. Distribution of Type I TA Systems among Different Species and Evolutionary Considerations 

It is difficult to analyze sequence conservation among type I toxins, because, in these systems, not the toxin protein, but the toxin mRNA and the antitoxin interact with each other. Mutations in the toxin mRNA will not affect base-pairing with the cognate antitoxin if the latter is transcribed from the complementary DNA strand. Such type I TA systems should be more prone to mutations than the type II TA systems. Conservation analysis (by Basic Local Alignment Search Tool (BLAST) search) of BsrG supported this hypothesis. Even closely related *Bacillus subtilis* strains display a variety of aa exchanges in BsrG, only maintaining the transmembrane domain. Thus, identification of homologous type I toxins is difficult.

Interestingly, BLAST search also identified conserved *bsrG*/SR4 loci in other *Bacillus* species, but some of them encoding BsrH/E instead of BsrG. This might be due to an exchange of the BsrG and the BsrE/H group toxin genes by mobile genetic elements via horizontal gene transfer. Moreover, the striking similarity of the secondary structures of *bsrG* mRNA and *bsrE* mRNA, as well as SR4 and SR5, implies a common ancestor of these two TA systems, although the toxicity and perhaps the mechanism of toxin action are different. BsrG encoded on prophage SPβ belongs to the same family as TxpA encoded on the skin element, and both cause lysis on agar plates in the absence of the antitoxin. By contrast, BsrE encoded by P6 is grouped with BsrH encoded on the skin element in one family, and only overexpression of *bsrE*—and most probably *bsrH*—is toxic.

The *txpA/bsrG*-like TA systems can only be found in a few *Bacillus* species, including *B. subtilis*, *B. halotolerans*, *B. amyloliquefaciens*, and also a *Paenibacillus* species. By contrast, *bsrE/bsrH*-like systems could be identified in a variety of *Bacillus, Lactobacillus*, *Halobacillus*, and *Enterococcus* species. Moreover, BsrE is also encoded on *B. subtilis* plasmid pBS72, but its effect on post-segregational killing is so far unknown. The genes encoding toxins YonT and YoyJ are also widespread in various *Bacillus* species, and sometimes truncated variants can be found, albeit mainly with conserved transmembrane (TM) domain regions.

Together, these findings provide evidence for an interspecies distribution of *B. subtilis* type I TA systems by mobile genetic elements such as phages or plasmids, for which they might have originally been addiction modules.

## 3. Type II Toxin-Antitoxin Systems

### 3.1. Overview of *B. subtilis* type II TA Systems

**Figure 4:**
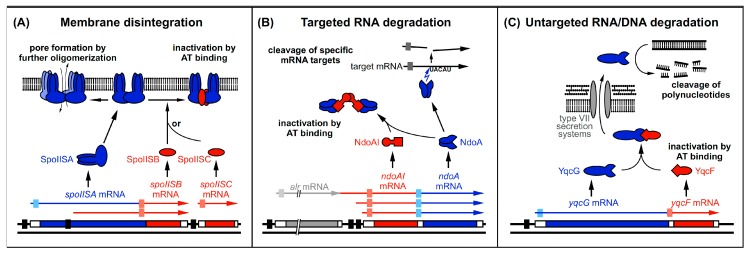
Three mechanisms of toxin and antitoxin action employed by *B. subtilis* type II TA systems. Black bars denote promoters. Toxins are drawn in blue and antitoxins in red. The toxin ORFs are represented by blue bars. Light-blue boxes indicate SD sequences. Antitoxin ORFs are represented by red bars and light-red boxes denote SD sequences. Arrows symbolize endoribonucleolytic activity. (**A**) SpoIISA toxin dimerizes in the membrane and is either inactivated by SpoIISB or SpoIISC or forms pores and causes membrane disintegration. (**B**) NdoA dimerizes and is either inactivated by NdoAI binding or cleaves RNAs upstream of single-stranded 5′–UACAU–3′ sequences. (**C**) YqcG is intracellularly inactivated by its antitoxin YqcF, predicted to be secreted by type VII secretion systems to be active outside the cell. There, it is proposed to cleave DNA to allow resorption of the cleavage products. The homologous toxins are proposed to cleave RNA.

The first type II TA system discovered in *Bacillus subtilis* was SpoIISA (YkaC)/SpoIISB/SpoIISC in 2001 [47]. In 2005, the NdoAI/NdoA (YdcE/YdcD; MazF/MazE) system was initially described as a homolog of MazF/MazE from *E. coli*, although a high similarity to *E. coli* plasmid-encoded PemK/PemI was already shown in 1998 [48]. In 2012, a set of six new and highly similar potential type II TA systems (YqcG/YqcF, YokI/YokJ, YobL/yobK, Yxid/YxxD, YeeF/YezG, and YwqJ/YwqK) were described and some of them further investigated [49]. Except for YqcG/YqcF and YokI/YokJ encoded on the skin element and on prophage SPβ, respectively, all other type II TA systems are encoded in the core chromosome. Table 2 and Figure 4 provide an overview of these type II TA systems.

*Bacillus subtilis* encodes a variety of further toxins or antimicrobial substances and corresponding immunity genes, e.g., SunA/SunI [50], WapA/WapI [51], SkfA, and SdpC [52]. However, most of their toxins are secreted, involved in contact-dependent growth inhibition (CDI), or do not match the criteria for typical type II toxins. Therefore, these systems are excluded here.

### 3.2. Toxins and Their Mode of Action

The toxin SpoIISA is a 248-aa protein with three N-terminal transmembrane (TM) domains (aa 1–87) and a cytoplasmic C-terminal domain (aa 88–248) [47,53]. Truncation studies showed that neither the N- nor the C-terminal domain alone can display the toxicity of the full-length protein [54]. Moreover, the TM domains themselves are involved in the toxicity mechanism. Deletion of TM3 or TM2/3, as well as exchange of all three TM domains via an alternative set of TM domains from SpoIIE, resulted in a non-functional toxin [53]. Also, a mutation (R38Q) in the cytosolic loop between TM1 and TM2 rendered SpoIISA non-toxic, most probably by preventing oligomerization of the N-terminal TM domains [47,53]. The cytosolic domain, resolved by crystallography, displays a globular GAF domain fold and enables SpoIISA to dimerize [55,56]. Furthermore, additional expression of the inactive SpoIISA_R38Q_ also inactivates the otherwise toxic SpoIISA, suggesting an oligomeric state for active SpoIISA [47]. Expression of *spoIISA* leads to disintegration of the cytoplasmic membrane, as well as detachment from and disruption of the cell wall [47].

Expression of *spoIISA* in *E. coli* showed a similar phenotype, suggesting a function directly in the plasma membrane and independent of further interaction partners [54]. It is assumed that SpoIISA forms holin-like pores in the plasma membrane [47], supported by the structural similarity of the N-terminal domain to class I holins and the observed phenotype. The effect of SpoIISA is bactericidal and irreversible [47,54]. 

The toxin NdoA is a 116-aa dimeric endoribonuclease that folds like the *E. coli* homolog MazF [57,58,59,60]. Upon dimerization, a groove is formed that binds extended single-stranded RNA (ssRNA) specifically at the sequence 5′–UACAU–3′ [60,61]. NdoA cleaves this sequence upstream of the 5′-U resulting in a 5′-hydroxyl and a 3′-phosphate group. Cleavage activity is independent of divalent cations [54]. Overexpression of *ndoA* in the absence of *ndoAI* inhibits growth of both *E. coli* and *B. subtilis*. Toxin action is rather bacteriostatic than bactericidal [57,61]. 

The toxins of the YqcG group are similar to each other. They consist of 531–669 aa with a well-conserved N-terminal and a more variable C-terminal domain (last 120–150 residues), both similar to contact-dependent growth inhibition (CDI) toxins in Gram-negative bacteria [49]. The C-terminal domain, most likely an HNH-fold, exerts endonuclease activity, which was shown for YobL, YxiD, and YqcG [49]. The differences in the C-terminal domains could cause slightly different nuclease specificities. While YobL, YxiD, and YqcG show RNase activity with a differing cleavage pattern in *E. coli* [49], in *B. subtilis*, YqcG displays exclusively DNase activity [62]. The N-terminal domain is supposed to be a secretion signal for type VII secretion systems, suggesting an extracellular function [49]. The C-terminal domains of YokI, YobL, YxiD, and YqcG exert toxicity in *E. coli* [49]. In *B. subtilis*, YqcG is bactericidal and rapidly kills the vast majority of cells. 

### 3.3. Mechanisms of Antitoxin Action

SpoIISA is inactivated by two similar antitoxins: SpoIISB (56 aa) and SpoIISC (45 aa) [47,56]. SpoIISB is a natively disordered protein able to bind to the C-terminal domains of SpoIISA dimers, resulting in a SpoIISA_2_·SpoIISB_2_ heterotetramer [55] (see Figure 4). SpoIISB wraps around the SpoIISA dimer forming comprehensive interactions with both SpoIISA subunits and covering wide surface areas [55]. Nearly all residues of SpoIISB are involved in the interaction with SpoIISA, resulting in a fast assembling and very stable complex of sub-nanomolar affinity [55]. Experiments with truncated SpoIISB showed that the C-terminal part is most crucial for antitoxin action and only the 12 N-terminal residues could be deleted without loss of function [55]. SpoIISC displays high similarity to SpoIISB. It also binds to SpoIISA and prevents toxicity [56], most likely acting in a similar way as SpoIISB. The detailed mechanism of action of the SpoIISB/C antitoxins is still unknown. There is evidence for the C-terminal domains of SpoIISA not only to dimerize, but also further multimerize to activate toxicity [56]. Subsequent SpoIISB binding to SpoIISA could prevent its further oligomerization by masking interaction sites. However, this still needs to be investigated.

NdoA is neutralized by binding of its antitoxin NdoAI. Two NdoAI proteins form a homodimer via their N-terminal RHH motifs. Each subunit displays an extended C-terminal helix that binds an NdoA dimer and blocks the RNA binding groove, resulting in an inactive and stable hexameric (NdoA)_2_–(NdoAI)_2_–(NdoA)_2_ complex [60] (see Figure 4). The homologous *E. coli* antitoxin MazE is not able to inactivate NdoA [61].

The toxicity of YqcG, YokI, YobL, and YxiD (and likely also YeeF and YwqJ) is counteracted by their cognate antitoxins [49]. Although not verified, the antitoxins likely bind at and block the active nucleolytic site. 

### 3.4. Expression and Physiological Role

Toxin action has to be tightly controlled by the cell to avoid undesired damage. Since toxin action is only aspired under very specific circumstances, most of the time, the antitoxin has to bind and neutralize the toxin. How this is achieved is detailed below.

In the *spoIISABC* system, three promoters could be identified (Figure 4). The first promoter P_A_ in front of the *spoIISA* gene yields a transcript covering at least *spoIISA* and *spoIISB*. A second promoter P_B_, about four times stronger than the first, is situated within the ORF of *spoIISA* and allows a strong additional transcription of *spoIISB* [47]. Both P_A_ and P_B_ enable basal transcription during growth and are strongly induced during sporulation [48]. At least P_A_ could be additionally regulated by the late sporulation sigma factor σ^K^ [63]. This expression pattern was further confirmed on protein level, showing a basal expression of *spoIISA* and a 10-fold induction at the onset of sporulation [64]. The third promoter P_C_ is less well investigated, but seems to be also induced during sporulation. All three promoters show enhanced activity under specific conditions; P_A_ is enhanced at ethanol stress, P_B_ during swarming and at high cell density, and P_C_ also during biofilm formation [46,56], likely to fine-tune toxin activation in response to environmental conditions. Whereas, for some type II TA systems, an autoregulatory feedback-loop was identified, there is no such evidence for *spoIISABC*. Furthermore, no data about SpoIISA/B/C protein stability are yet available. However, the unstructured fold of unbound antitoxin is an indication for a relative instability of SpoIISB (and most likely also SpoIISC).

The physiological role of the *spoIISABC* system remains unknown. Although toxin expression seems to be highest during sporulation, antitoxin expression is also elevated at the same time. One study used a combined *ndoA/spoIISA* deletion strain and observed 25% less programmed cell death and an altered morphology in biofilms [65]. Although this was a combined deletion of two toxin genes, this is the only effect found so far.

The *ndoA/ndoAI* system is transcribed as a bicistronic *ndoAI-ndoA* mRNA under control of one or two *ndoAI* promoter(s) which are yet to be further characterized. As the gene *alr* located upstream of *ndoAI* lacks a detectable terminator, read-through transcripts from the *alr* promoter cannot be excluded [46] (see Figure 4). While many homologous systems like *E. coli mazE/mazF* are autoregulated [66], this is not yet shown for *ndoAI/ndoA* in *B. subtilis*, although a palindromic sequence at the *ndoAI* promoter suggests autoregulation [57]. Nucleolytic activity of NdoA was initially detected in *B. subtilis* crude extracts [57], suggesting a faint constitutive activity and perhaps additional activation under specific conditions.

The physiological role of *mazF/mazE* systems in general is under debate. Notably, in *E. coli*, where the system is well characterized, a role in stress response, heterogeneity, and persistence is discussed (reviewed in Reference [67]). Since NdoA cleaves a specific pentad sequence, a role in expression regulation of specific mRNAs is conceivable, taking into account that NdoA is not directly toxic to the cell. The 5′–UACAU–3′ pentad sequence was found to be enriched in genes involved in secondary metabolite biosynthesis [61]. In the absence of *ndoA,* cells were more resistant against antimicrobials and some environmental stressors like hydrogen peroxide, but also more sensitive to heat and high ultraviolet (UV) irradiation [68], suggesting a role in programmed cell death or stress response regulation. An “extracellular death factor”, the hexapeptide RGQQNE, produced by *B. subtilis* cells as a quorum-sensing molecule, activates NdoA by blocking antitoxin, but not RNA binding [69], further supporting a role during stress. Moreover, *ndoA* deficiency suppresses sporulation [68]. *E. coli* MazF generates leaderless mRNAs, as well as ribosomes with cleaved 16S rRNAs, thus establishing a modified translational machinery to expedite expression of specific genes [70]. However, *B. subtilis* 16S rRNA lacks a recognition site for NdoA, rendering this mechanism unlikely.

Transcription of all six *yqcG* homologs is highly similar and identical to their cognate antitoxin genes. Expression of both toxins and antitoxins is elevated during sporulation, under starvation, and at high cell density [46]. Additionally, *yqcG* transcription during sporulation was shown to be dependent on Spo0A via the SinI/SinR pathway and further via DegU [71,72]. Considering genomic and translational similarities, this is likely also true for the other homologous systems. In the case of *yobL*/*yobK*, *yeeF*/*yezG*, and *yqcG*/*yqcF,* toxin and antitoxin genes are transcribed bicistronically. By contrast, *yxiD*/*yxxD*, *yokI*/*yokJ*, and *ywqJ*/*ywqK* are located in in larger operons and are, therefore, translated from multicistronic transcripts [46].

Most likely, these six highly similar systems fulfil comparable functions, although their C-terminal toxin domains differ. The shared putative N-terminal secretion signal implies a function outside of the cell. YqcG was shown to eliminate defective cells and to reduce the number of living cells in starved cultures and biofilms [72]. This could save resources and enable a part of the population to survive longer under harsh conditions. Alternatively, the systems could provide an advantage in competitive growth by inhibiting other strains and/or provide nutrients by degrading cellular debris [49]. Both functions could benefit from several slightly different copies of the toxins. 

### 3.5. Distribution of Type II TA Systems among Different Species and Evolutionary Considerations

Like type I TA systems, type II TA systems are thought to originate from plasmids, phages, or other mobile genetic elements where they function in post-segregational killing. However, type II TA systems are also found in well-conserved regions of the core genome, where they were not eliminated during evolution and, most likely, adopted by the cells for own regulatory fine-tuning.

The *spoIISABC* system is part of the core genome in many *Bacillus* species and also found in some non-*Bacillus* species, likely adopted by horizontal gene transfer [73]. However, some relatives secondarily lost the system during evolution, whereas a few even acquired a second copy [73]. In *Bacillus subtilis* and close relatives, the gene locus is adjacent to the PBSX prophage, but does not seem to be involved in phage maintenance. A few other homologs were also found on plasmids [73]. However, at least in the heterologous *E. coli*, the system was not able to maintain plasmid stability by post-segregational killing [61]. All known *spoIISABC* systems display a high dissimilarity with only four invariant and eight conserved SpoIISA residues [73]. The second antitoxin is a result of a gene duplication that could even have happened twice and independently in two lineages [73].

The *ndoAI/ndoA* (*mazE/mazF*) system is widely distributed among Gram-positive and Gram-negative bacteria. However, *B. subtilis ndoA* shares much more similarity to *mazF* homologs of other Gram-positive bacteria than to those of Gram-negative bacteria [61]. In addition, the target specificity differs. While *E. coli* MazF cleaves 5′–ACA–3′ sequences [74], *B. subtilis* NdoA needs a more specific and less abundant 5′–UACAU–3′ sequence [61]. Despite huge differences in aa sequence, folding structure, and endonucleolytic activity of MazF, R1 plasmid-encoded toxin Kid, F plasmid-encoded CcdB from *E. coli*, and NdoA from *B. subtilis* are well conserved [59], suggesting the same evolutionary origin.

**Figure 5:**
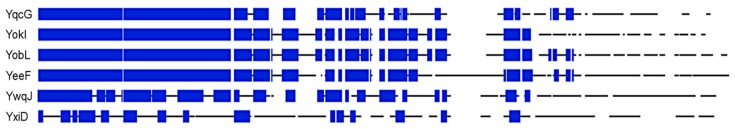
Multiple alignment of aa sequences of YqcG homologs from *B. subtilis*. Alignment was performed using a BLOSUM 62 scoring matrix and full-length protein sequences. Sequence stretches are represented as black lines and white gaps are gaps in the alignment. Stretches of at least 5 aa with at least 60% similarity are depicted as blue boxes.

The six YqcG homologs are very similar and most likely have a common ancestor (see Figure 5). Comparable genes can be found in various *Bacillus* species. In addition, similarities to the widespread CDI genes from Gram-negative bacteria are apparent. Both toxin domains, the well-conserved putative N-terminal translocation signal and the toxic C-terminal domain harboring nuclease activity, are similar (reviewed in Reference [75]). However, the *Bacillus subtilis* genes do not share the central spacer domain, presumably required to span the distance to another cell to realize CDI action. Interestingly the *wapA* gene of *Bacillus subtilis* resembles such a full-length CDI [51], but the sequence identity to the *yqcG* family members is relatively low.

## 4. Concluding Remarks

To date, only type I and type II TA systems were discovered in *B. subtilis* and investigated to some degree. Although valuable information about toxin action could be obtained for BsrG, the targets of all type I toxins are still unknown. On the one hand, they might be encoded by essential genes as seems to be the case for BsrG. On the other hand, it is challenging to purify sufficient amounts of these membrane-bound toxins for biochemical studies, even with tightly controlled expression systems, as only a few molecules suffice to kill the host cells. Furthermore, experiments are needed to elucidate whether the additional nine potential systems, whose putative toxins were grouped into the YhzE family [9], are indeed type I TA systems. 

For the type II systems, the picture is different. SpoIISA seems to act like a phage holin, although more data, e.g., on its effects on the membrane potential, are needed to confirm this assumption. Whereas NdoA that resembles *E. coli* MazF is known to act as interferase to degrade specific mRNAs, its physiological role is still unknown. YqcG and some of its homologs act as DNases and RNases, but their sequence specificity has to be analyzed and their location confirmed. Although the crystal structures of SpoIISA and NdoA were solved, information regarding regulatory and physiological function is sparse and hard to obtain. Currently, we cannot exclude that *B. subtilis* and other *Bacillus* species might also encode type III, IV, or V TA systems. Moreover, more type I and type II systems or even combined systems could exist. More bioinformatics and subsequent experimental work is required to answer these questions.

## Figures and Tables

**Table 1:** Overview of type I toxin-antitoxin (TA) systems from *Bacillus subtilis:* characteristics, mechanism of antitoxin action, RNases involved in degradation of toxin messenger RNA (mRNA) and RNA antitoxin, and regulation of expression.

TA System	Location on Prophage	AT (nt)	T RNA (nt)	Mode of AT Action	RNases Cleaving T/AT RNA	T (aa)	Regulation/Peculiarity
*txpA*/RatA	skin	222	270	RD	III *, P/III	59	glucose-dependent
*bsrG*/SR4	SPβ	180	294	TI + RD	III, Y, R/III, R, Y, J1, P	38	temperature-dependent
*bsrE*/SR5	P6	163	255–260	RD	III *, Y, P/III, J1, P	30	multistress-responsive
*yonT-yoyJ/*SR6	SPβ	100/215 ^a^	>450	RD *yonT* TI *yoyJ*	III */III	58	multistress-responsive, SR6 unusually stable
*bsrH*/as-bsrH	skin	200	285	RD ?	J1/n.d.	29	multistress-responsive, glucose-dependent

AT, antitoxin; T, toxin; TI, translational inhibition; RD, promotion of toxin mRNA degradation; aa, amino acid; ?, proposed but not experimentally shown. ^a^, longer SR6 species due to read-through of SR6 terminator. III, RNase III; Y, RNase Y; R, RNase R; J1, RNase J1; P, PnpA. * Essential for inhibition by the antitoxin; n.d., not determined. For further details, see text.

**Table 2:** Overview of type II TA systems from *B. subtilis:* toxin and antitoxin properties, toxin target, and antitoxin necessity.

Toxin (T)	Antitoxin (AT)	T (aa)	AT (aa)	T Target	AT Essential
SpoIISA (YkaC)	SpoIISB	248	56	plasma	no
	SpoIISC	248	45	membrane	no
NdoA (YdcE, MazF)	NdoAI (YdcD, MazE)	116	93	5′–UACAU RNA	not determined
YqcG	YqcF	531	192	DNA	yes
YokI	YokJ	571	165	DNA or RNA	no
YobL	YobK	600	152	RNA	no
YxiD	YxxD	569	147	RNA	yes
YeeF	YezG	669	151	DNA or RNA	yes
YwqJ	YwqK	602	154	DNA or RNA	no

## Abbreviations

aa	amino acid
bp	base-pair
CDI	contact-dependent growth inhibition
L	loop
nt	nucleotide
RBS	ribosome binding site
SD	Shine Dalgarno sequence
SL	stem-loop
sRNA	small regulatory RNA
ssRNA	single stranded RNA
TA	toxin–antitoxin
